# New Strategy to Cope with Common Fishery Policy Landing Obligation: Collagen Extraction from Skins and Bones of Undersized Hake (*Merluccius merluccius*)

**DOI:** 10.3390/polym11091485

**Published:** 2019-09-11

**Authors:** María Blanco, Carmen G. Sotelo, Ricardo I. Pérez-Martín

**Affiliations:** Department of Food Biochemistry, Instituto de Investigaciones Marinas (CSIC), Eduardo Cabello 6, 36208 Vigo, Spain; carmen@iim.csic.es (C.G.S); ricardo@iim.csic.es (R.I.P.-M.)

**Keywords:** landing obligation, fish discards, skin and bone valorization, acid soluble collagen, pepsin soluble collagen

## Abstract

In order to promote sustainable fishing practices within European fishing fleets and to avoid the large waste of valuable fish biomass through the practice of fish discarding, the new reform of the common fisheries policy includes the obligation of landing all species under total allowable catch (TAC) regulations. The new policy also prohibits the use of specimens under minimum conservation reference size for direct human consumption. In this context, it is necessary to find new uses for undersized fish, which might help to alleviate the costs associated with the landing obligation but without prompting the creation of a market. European hake (EH) (*Merluccius merluccius*), which is one of the most important commercial fish species for the Spanish fishing industry, with a total TAC for 2018 of 37,423 t, is used for this study. Consistent with the current policy framework and taking into account the commercial importance of this species, the aim of this work is to study a new strategy for the extraction of collagen from the skin and bone fraction of *Merluccius merluccius* undersized discards. Three collagen fractions are successfully isolated for the first time from the skin of *M. merluccius* skin and bone discarded raw material: acid-soluble collagen (ASC) fraction 1 and pepsin-soluble collagen (PSC) fraction 2 from the skin and ASC fraction 3 from bones. The total collagen yield of the process is 13.55 ± 3.18% in a dry basis (g collagen/100 g of skin and bone fraction (SBF)) and 47.80 ± 9.83% (g collagen/100 g of collagen determined by the hydroxyproline content in SBF). The three fractions are further characterized by using different physical and chemical analysis techniques, with the conclusion drawn that the triple helix structure is preserved in the three fractions, although ASC fractions (F1 and F3) present more or stronger hydrogen bonds than the PSC fraction (F2). With the process herein presented, deboned and skinned hake specimens could represent an interesting source of high quality type I collagen, which could be useful as a raw material for the biomedical, cosmetic, and nutraceutical industries.

## 1. Introduction

In order to promote environmentally, economically, and socially sustainable fishing practices within European fishing fleets, the new reform of the common fisheries policy includes the obligation of landing all commercially exploited species under the total allowable catch (TAC) regulations, aiming to avoid the large waste of valuable fish biomass through the practice of fish discarding [1]). The new policy also prohibits the use of catches below the minimum conservation reference size (MCRS) for direct human consumption. In this context, it is necessary to find new uses for undersized fish, which might contribute to the alleviation of costs associated with the landing obligation but without prompting the creation of a market. If fishers do not receive any compensation for the costs involved in landing all the catch, the successful implementation of the new regulation might be at risk. Although the incentives should be focused on minimizing and avoiding unwanted catches, for example, specimens of species below the MCRS, it is expected that few of those undersized catches still occur depending on several factors such as type of fishery/gear, area, or time of the year.

The final objectives of the new landing obligation are also in line with other two EU strategies: blue growth and the 2020 EU strategy, which are both involved in the development of sustainable socioeconomic and environmental growth in the marine and maritime EU region. One of the components of blue growth strategy is focused on the “blue biotechnology” covering the biotechnological transformation of raw marine materials for the isolation/production of molecules that could be used in a diverse array of applications, including antioxidants, antiinflamatories, antifoulings, biomarkers, etc., which might finally foster the development of innovative markets for those compounds contributing to the objectives of the EU strategies.

The valorization strategies for fish discards and fish by-products with the final purpose of obtaining valuable bio-compounds useful for different applications include a wide range of processes such the production of protein hydrolysates, bioactive peptides, collagen/gelatin, peptones, chondroitin sulfate, and chitin [2,3,4,5]. Nowadays, there is a growing demand for marine-derived collagen as an alternative to mammalian sources due to religious or disease concerns that could be used for different applications, including cosmetics, tissue engineering, and other biomedical and pharmaceutical uses [6,7].

The collagen-containing materials in teleost fish, mainly skin and bones, could account for as much as 22.5% of the total fish weight [8,9]. Such an important percentage makes the skin and bone fraction of discarded fish an excellent raw material for the extraction of collagen. Collagen, the main structural protein in connective tissue, has a particular heterotrimeric structure that has been previously described in several species [10,11,12]. However, most studies on collagen extraction use only skin as raw material [11,13,14]. Some studies have also described the extraction and properties of collagen obtained from other sources such as bone, scales, or fins [15,16]; however, this is the first time, as far as the authors know, in which a mixture of skin and bones obtained by mechanical deboning has been used to extract collagen from European hake (*Merluccius merluccius*) undersized (≤27 cm) specimens.

European hake has been selected, as it is one of the most valued and commercialized fresh products consumed in EU countries; in 2017 almost 112.000 tones were consumed, with a value of 851 million € [17]. Regarding Spanish fleets, this species is also of high importance with a total TAC in 2018 of 37.423 tones. Discard rates of hake specimens below the minimum landing size (MLS) have been reported to be high, both in the ICES regions and Mediterranean waters [18]. For the otter bottom trawlers targeting demersal species in north Spanish Iberian Waters, ICES Divisions VIIIc and IXa, the estimated proportion of undersized hake discarded were 611 Tm (45% of hake captures), 214 Tm (18% of hake captures) and 698 Tm (97.8% of hake captures) for 2011, 2012, and 2013, respectively [19]. In order to comply with the new reform of the common fisheries policy, which has been fully implemented for all regulated commercial species on 1st January 2019, and bearing in mind the growing demand for marine derived collagens, a new strategy for the extraction of collagen from the skin and bone fraction of *M. merluccius* undersized discards is proposed.

## 2. Material and Methods

### 2.1. Biological Samples

34 kg of European hake (*M. merluccius*) specimens were caught by a commercial trawler (Ría de Marín) on the coast of northern Portugal (42°N19′09W22′ and 245 m depth). To estimate the average weight and size of the specimens, sample was divided into three batches, and from each of them twenty specimens were randomly selected. The individuals not used for the size/weight sampling were manually headed and gutted and used for the mechanical separation of muscle from skin and bones using a fish deboned (Josmar S.L). The skin and bone fraction (SBF) were then homogenized using a pilot scale cutter (Fatosa, Spain), divided into batches of 200 g, and kept frozen at −20 °C until they were used for the collagen extraction.

#### 2.1.1. Proximate Composition of SBF

The chemical composition of SBF was evaluated in terms of triplicate analyzing moisture, crude protein, lipids, and ash content. Total nitrogen was determined with the Kjeldahl method [20] in a DigiPREP HT digestor (SCP Science, Baie-d’Urfe, Qc, Canada), DigiPREP 500 fully automatic steam distillation (SCP Science, Baie-d’Urfe, Qc, Canada), and TitroLine easy tritation unit (Schoot, Mainz, Germany), and crude protein content was calculated as total nitrogen multiplied by 6.25. Fat content was determined by the method of Bligh and Dyer [21]. Moisture was determined after heating the sample at 105 °C for 24 h, and ash content was determined after heating the sample for 24 h at 550 °C [20].

#### 2.1.2. Hydroxyproline Content

Seventy mg of dried and grounded raw material (SBF) was introduced in hydrolysis microwave tubes, and 20 mL of 6M HCl was added. Hydrolysis was performed in a microwave (speed wave MWS-2) (Berghof GmbH, Eningen, Germany) at 150 °C for 90 min at 70% power. Once the hydrolysis step was finished, samples were allowed to cool down to room temperature and were made up to a known volume with 6 M HCl. Four hundred µL of this solution was transferred to glass vials and left to dry in vacuum dessicator at 60 °C in the presence of solid NaOH, after drawing air for four days. The resulting dry matter was suspended in 8 mL of buffer (0.13M citric acid, 0.75% glacial acetic acid, 0.6 M sodium acetate, 0.15 M sodium hydroxide, and 20.13% *n*-propanol; pH was adjusted to 6.5 with 0.2 M NaOH, and volume was brought to 660 mL with distilled water).

### 2.2. Collagen Extraction and Characterization

**Figure 1:**
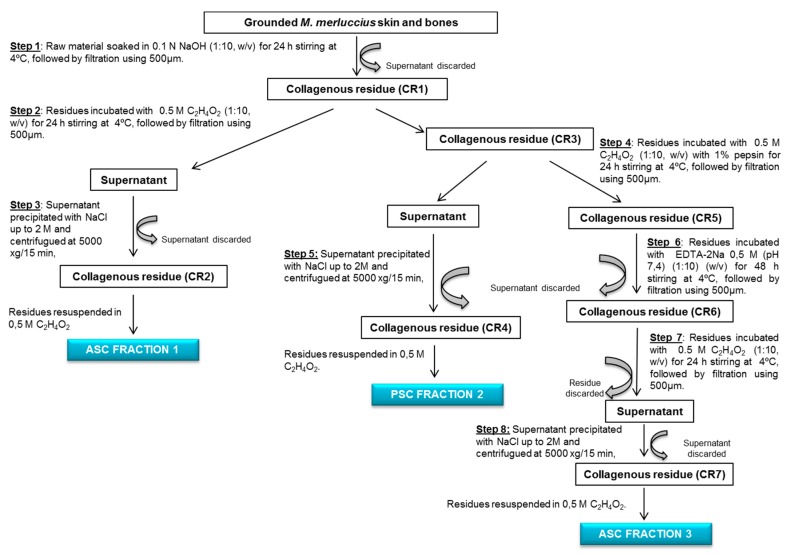
Diagram showing the operations employed for the recovery of three collagen fractions from *Merluccius merluccius* skin and bones: ASC (fraction 1) and PSC (fraction 2) from the skin and acid-soluble collagen (ASC) from bones (Fraction 3).

Three collagen fractions were extracted from grounded raw material (SBF) following the scheme shown in Figure 1. All procedures were performed at 4 °C. SBF was first treated with 0.1 N NaOH (1:10, *w/v*) for 24 h with continuous stirring followed by filtration through 500 µm membrane. The supernatant was discarded, while the collagenous residue (CR1) was incubated with 0.5 M acetic acid (1:10, *w/v*) for 24 h with continuous stirring, followed by filtration through 500 µm membrane. The supernatant was salted-out by adding NaCl (final concentration of 2 M) and centrifuged at 5000× *g* 15 min. The supernatant was discarded, and the collagenous residue (CR2) was resuspended in 0.5 M acetic acid constituting the acid soluble collage ASC fraction 1 (ASC F1). The skin and bones collagenous residue (CR3) obtained after step 2 were incubated with 0.5 M acetic acid (1:10, *w/v*) with 1% pepsin for 24 h with continuous stirring followed by filtration using 500 µm. The supernatant obtained was salted-out by adding NaCl (final concentration 2 M) and centrifuged at 5000× *g* 15 min. The supernatant was discarded, and the collagenous residue (CR4) was resuspended in 0.5 M acetic acid constituting the Pepsin soluble collagen fraction 2 (PSC F2). The collagenous residue obtained after step 4 was incubated with 0.5 M EDTA-2Na (pH 7.4) (1:10, *w/v*) for 48 h with continuous stirring followed by filtration using 500 µm membrane. The supernatant was discarded, and the collagenous residue (CR6) was incubated with 0.5 M acetic acid (1:10, *w/v*) for 24 h with continuous stirring followed by filtration using a 500 µm membrane. Then, the residue was discarded and the supernatant salted-out using NaCl (final concentration of 2 M) and centrifuged at 5000× *g* 15 min. The collagenous residue (CR7) was resuspended in 0.5 M acetic acid, constituting the ASC fraction 3 (F3). F1, F2, and F3 collagens solutions were dialysed, freeze-dried, and maintained under vacuum-drying.

The three collagen isolated fractions were further characterized by using different physical and chemical analysis techniques, with the objective of evaluating the collagen structure to assess its posterior potential use.

#### 2.2.1. Molecular Weight Determination of Collagen by GPC

The size exclusion chromatography (SEC) measurements were carried out in an Agilent 1260 HPLC consisting of a G1311B quaternary pump, a G1329B injector, a G1316A column oven, a G1362A refractive index (RI), and a dual-angle static light scattering (DALS) G7800A detector. For chromatographic separations, four columns were used (PSS, Germany): Proteema precolumn (5 µm, 8, 50 mm), Proteema 1000 Å (5 µm, 8, 300 mm), Proteema 300 Å (5 µm, 8, 300 mm), and Proteema 100 Å (5 µm, 8, 300 mm). For sample preparation, lyophilized collagen (F1, F2, and F3) was dissolved in the mobile phase (0.15 M sodium acetate, 0.2 M acetic acid, and pH 4.5) (1 mg/mL) and filtered through a PTFE 0.2 µm membrane filter. A total of 100 µL of the filtered solution was injected and chromatographed at an eluent flow rate of 0.5 mL/min using an isocratic elution profile. Column oven was kept at 20 °C, RID at 35 °C, and DALS at 30 °C. DALS detector was calibrated with a polyethylene oxide standard (PEO) (PSS, Germany) of 106 kDa (Mp) and polydispersity index 1.05. Refractive index increments (dn/dc) were adopted from Meyers and Morgenstern [22]. Data were analyzed using Agilent GPC/SEC software A.02.01.

#### 2.2.2. Circular Dichroism

Circular dichroism (CD) measurements of three collagen isolated fractions (F1, F2, and F3) were performed using a Jasco J-1100 spectropolarimeter (Jasco, UK). The used wavelength range for measurements was 180–240 nm at a scan rate of 100 nm/min. Measures were done in triplicate at 18 °C. Data were analyzed using Spectra Manager software (Jasco, UK).

#### 2.2.3. X-Ray Diffraction (XRD)

XRD measurements were obtained using a diffractometer system (XPERT-PRO, Panalytical) equipped with Cu*Kα* radiation, produced at 40 kV and 30 mA. Data sets were collected in the 2*θ* range of 40° to 80° with a step size of 0.02° and a scanning speed of 1s at 4°/min. The average crystallite size was estimated with the Bragg equation (Equation (2)): d (Å) = Cu (*λKα* = 1.5406 Å).

#### 2.2.4. Fourier-Transform Infrared (FTIR) Spectroscopy

The infrared spectra of three collagen isolated fractions (F1, F2, and F3) were obtained with a FTIR Nicolet 6700 spectrometer (Thermo Scientific, Waltham, MA, USA), coupled to an Smart Orbit (with a diamond crystal), in the spectral region of 400–4000 cm^−1^ with resolution of 4 cm^−1^, an average of 32 scans. A deuterated triglycine sulfate (DTGS) detector was used together with a germanium-coated potassium bromide beam splitter. Samples were directly measured at ambient temperature by attenuated total reflection (ATR) without any further preparation.

#### 2.2.5. Amino Acid Composition

Amino acids were determined in the three lyophilized fractions (F1, F2, andF3). For this purpose, an acid hydrolysis was developed using 6 N HCl containing 0.1% phenol under an inert atmosphere by heating at 110 °C for 24 h. Then, HCl was removed by vacuum. The hydrolysate was resuspended in 20–50 µL of 0.2 M sodium citrate buffer (pH 2.2), to which a known amount of norleucine was added as an internal standard and applied to an automated amino acid analyzer (Biochrom30 Amino Acid Analyzer, Biochrom, UK). Differences regarding the amino acid content between the three fractions were tested by one-way analysis of variance (ANOVA). A Post hoc comparison test was applied. Significance levels were set at *p* ≤ 0.05. Statistical tests were performed with IBM SPSS 25 (IB; Corporation, Armonk, NY, USA).

#### 2.2.6. SDS-PAGE

The protein pattern of F1, F2, and F3 fractions of *M. merluccius* collagen samples was analyzed using sodium dodecylsulfate poly-acrylamide gel electrophoresis (SDS-PAGE) according to the procedure described in Laemmli [23]. Samples (1/mg/mL) were prepared in sample buffer containing 10.52% glycerol, 21% Sodium Dodecyl Sulfate (SDS) (10%), 0.63% Dithiothreitol (DTT), and 0.5 M Tris-HCl (pH 6.8) and heated for 5 min at 100 °C. An aliquot (8 µL) of this mixture was applied to each well in 7% polyacrylamide separating gels (100 mm × 750 mm × 0.75 mm) and subjected to electrophoresis at 20 mA using a mini-protean II cell (Bio-Rad, Hercules, CA, USA). After, electrophoresis gels were stained with 0.04% Coomassie Blue in 25% ethanol (*v*/*v*) and 8% acetic acid (*v*/*v*) for 2 h. Excess stain was removed with several washes of destaining solution (25% ethanol (*v*/*v*), 8% acetic acid (*v*/*v*)). Molecular weights of collagen subunits were estimated using two wide range molecular weight standards (Amresco and Fermentas).

## 3. Results and Discussion

### 3.1. Characterization of Hake Specimens

**Figure 2:**
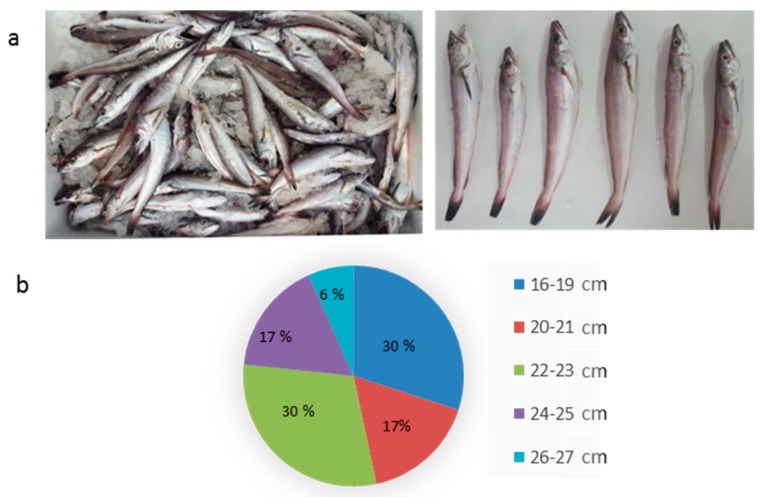
Photograph of some of the hake specimens used in the present study (**a**). Percentage of hake individuals of each size is shown in each sector of the diagram; (**b**) along with the corresponding size ranges (cm), which are indicated in the legend.

Hake (*Merluccius merluccius*) specimens ranging from 16 cm up to 26 cm were captured by a commercial trawler. Figure 2a shows the appearance of the hakes used in the study, where it can be observed that small individuals were mixed together with bigger ones. Figure 2b shows the frequency of sizes of a representation of a sample of 20 individuals, showing that the sample was rather heterogeneous, with two sizes most represented (60% both together): smaller individuals from 16 cm up to 19 cm and individuals ranged 22–23 cm long. The average length was of 21.23 cm ± 2.412, and average weight was 66.48 g ± 21.580.

Minimum legal size for *Merluccius merluccius* captured in Atlantic Ocean waters, like the ones in this study, is of 27 cm [24]. Therefore, all the individuals captured are under this legal size and therefore must comply with the landing obligation regulations [1]. The landing obligation of this biomass stresses the need of diminishing the costs associated with its capture and transportation to land, and this can only be done by finding some valorization strategies that help the industry to cope with the costs of the discard ban.

Another important aspect to consider is the legal requirement to land this type of fish, which has limited uses, with the objective to avoid creating markers for undersized fish [25].

### 3.2. Mechanical Separation of Skin and Bone Fraction (SBF): Yields and Chemical Characterization

**Figure 3:**
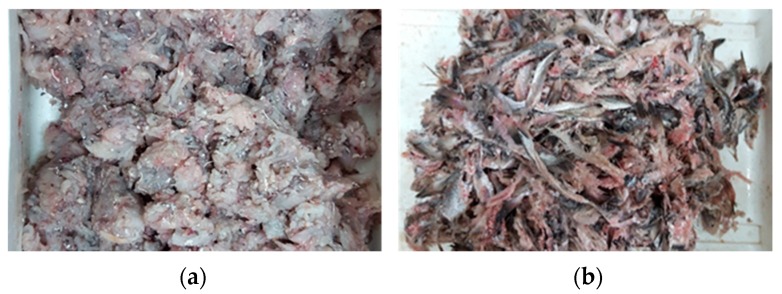
Fractions obtained after the mechanical separation of headed and gutted individuals of undersized hake: (**a**) muscle fraction and (**b**) skin and bone fraction.

In order to design an adequate valorization strategy for hake individuals below the minimum landing size (≤27 cm), the study of the yield and chemical characterization of the raw material susceptible to valorize is of great importance. Individuals were manually headed and gutted, rendering the following percentages: 22.06% of heads, 5.90% of viscera, and 68.80% of bodies. These values were similar to those found in similar species [26]. This high percentage of headed and gutted fish makes this raw material a promising source for valorization. After the mechanical separation of skin and bone fraction from the muscle of headed and gutted specimens, the yields were as follows: minced muscle represented 76.25% (Figure 3a), whereas skin and bones represented 18.6% (Figure 3b). The muscle fraction could only be used, in this case, to obtain a high-quality fish protein hydrolysate since the direct human consumption of undersized specimens is not permitted in the new LO regulation. As the valorization of the muscle fraction of different species for non-food purposes has been previously studied [2,4,5], the objectives of this study are focused on the potential valorization of the SBF fraction by the extraction of collagen and its characterization. The SBF is rather heterogeneous, and bones and fins are clearly observed in the mixture.

The mixture of skin and bones were mechanically grinded, and the chemical composition of the resulting material was determined. The mean ± SDs for the water, lipid, ash, and protein contents (in wet basis) of the mixture were of 74.38% ± 0.125, 2.14% ± 0.192, 6.65% ± 0.062, and 17.20% ± 0.112, respectively. The ash content was relatively high, reflecting the presence of minerals from the bones and scales in the mixture. Hydroxyproline content was also determined in the mixture for estimating the potential collagen content in the raw material (SBF), assuming that all HPro content of SBF was due to collagen and considering that the ratio of HPro in collagen was 12.5 g of HPro/100 g of collagen [27]. The obtained value was of 15.32% ± 0.381 of collagen on a dry basis. This value collagen was lower than that reported previously in the skin of different teleost or chondrychtyes species [2,3]. These differences could be due to the raw material used for collagen extraction, as the bones included in the skin and bone fraction presented a lower collagen content compared than that of the skin.

### 3.3. Collagen Extraction: Protein and Collagen Yields

**Figure 4:**
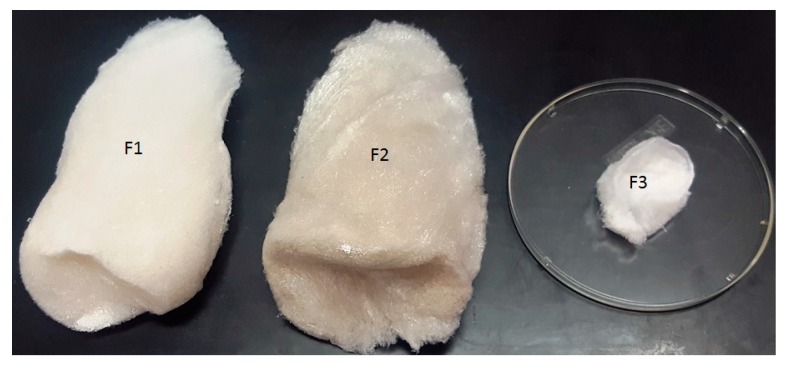
A representation of the three collagen fractions obtained from a mixture of *M. merluccius* skin and bones. ASC from the skin (F1), pepsin-soluble collagen (PSC) from the skin (F2), and ASC from the bone (F3).

As far as the authors know, this is the first study evaluating the possibility of extracting collagen from a mixture of skin and bones obtained from the mechanical separation of the muscle using undersized hake specimens. This is also the first time in which three collagen fractions have been recovered from the SBF (Figure 4): an acid-soluble fraction (F1) and a pepsin soluble fraction (F2) from the SBF; the remaining residue of the process, which consists mainly of bones, produces another acid soluble fraction (F3) (Figure 4).

**Figure 5:**
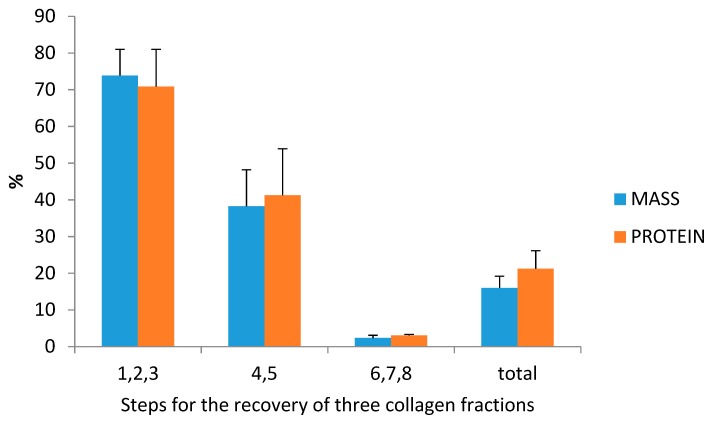
Percentages of retained mass and protein within the different steps involved in the recovery of collagen from the skin and bone fraction (SBF) of *M. merluccius* undersized specimens. Values are means and standard deviations obtained from three different extraction procedures. The different steps involved in the recovery of the collagen fractions are explained in Figure 1.

The yield of dry mass and collagen retained in each extraction step (F1, F2, and F3) was analyzed (Figure 5). The steps 1, 2, and 3 from the Figure 1 retained the highest amount of mass (73.84% ± 7.15), whereas the steps 4 and 5 (pepsin solubilization step) retained the highest amount of protein (70.86% ± 10.14). The lowest retention efficiency was found in the steps 6, 7, and 8 for both mass (2.33% ± 0.76) and protein (3.06% ± 0.29). Overall yields of the process were moderately low; the total amount of retained mass was of 15.98% ± 3.19, while retained protein was 21.24% ± 4.87. Therefore, although both acetic acid and pepsin solubilization steps solubilize proteins were present in the skin, not all the protein present could be solubilized using this approach. Furthermore, the solubilization efficiency of proteins in the case of bones was rather low, probably due to covalent links of proteins with other bone molecules, which were not broken with an acid treatment.

The total collagen yield of the process was 13.55 ± 3.18% in a dry bassis (g collagen/100 g of SBF) and 47.80 ± 9.83% (g collagen/100 g of collagen determined by the hydroxyproline content in SBF). Similar percentages of ASC (F1) and PSC (F2) from the skin were obtained during the process—45% and 54%, respectively—whereas the percentage of ASC from bones (F3) represented less than 1% of the total collagen obtained.

### 3.4. Characterization of Collagen

The three collagen fractions obtained from skin and bones were further characterized using different physical and chemical analysis techniques. The objective was to evaluate the structure of the obtained collagen to assess its potential uses.

#### 3.4.1. Molecular Weight of Collagen

**Figure 6:**
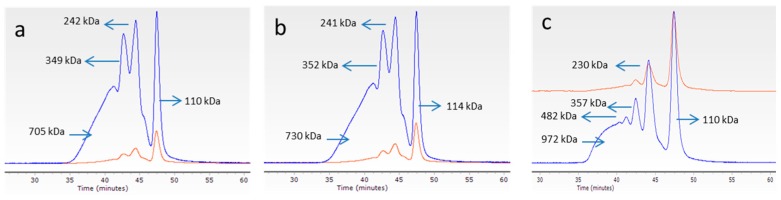
Weight average molecular weights (*M*_w_) of *M. merluccius* collagen fractions (**a**) F1, (**b**) F2, and (**c**) F3. Blue line: 90° light-scattering signal (LS); red line: refractive index signal (RI).

The weight average molecular weights (*M*_w_) of *M. merluccius* collagen fractions (F1, F2, and F3) are shown in Figure 6. GPC chromatograms of F1, F2, and F3 fractions (a, b, and c, respectively) are very similar, showing a clear peak with molecular weight of 110 kDa for F1 and F3 and 114 kDa for F2, which can be assigned to the *α*-chains (monomer). Chromatograms also show the presence of the *β* component (dimer) with molecular weights of 242 kDa, 241 kDa, and 230 kDa for F1, F2, and F3, and *γ* components (trimer) with molecular weights of 349 kDa, 352 kDa, and 357 kDa for F1, F2, and F3, respectively. These values agree with values reported for collagens extracted from other fish species [22] and indicate that a typical type I collagen is present in these three fractions.

#### 3.4.2. Amino Acid Content

Table 1 shows the amino acid composition of the three collagen fractions obtained from the SBF. Amino acid composition has not been reported previously in the three collagen fractions obtained in this study. No significant differences were found between the three fractions. Glycine was the most abundant amino acid in the three fractions, representing one third of the total amino acid content. Hydroxyproline, alanine, proline, and glutamine were the most common amino acids found in the three fractions, which agrees with results found in collagen extracted from different marine species [10,28]. The degree of hydroxylation of proline was 36%, 35%, and 37% for F1, F2, and F3 fractions, respectively. These results were lower than those obtained in other teleost and in elasmobranch [10,29], indicating the low stability of the triple helix in this species.

#### 3.4.3. FTIR

**Figure 7:**
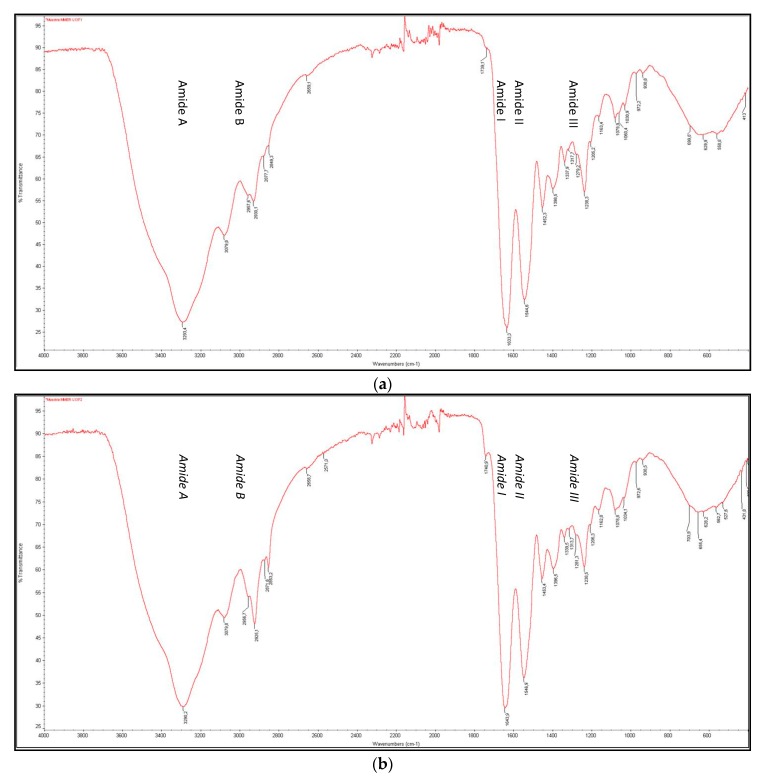

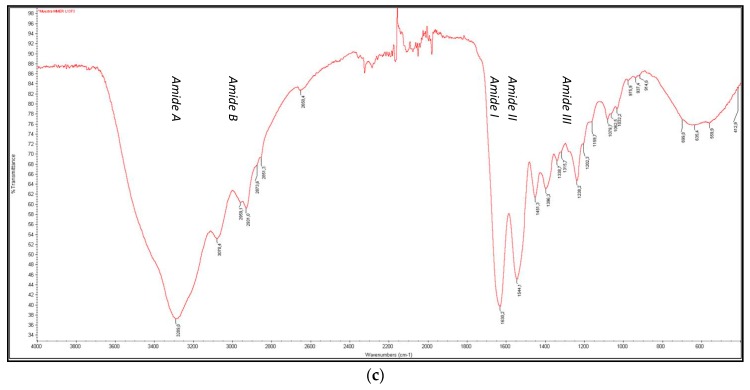
Fourier-transform infrared (FTIR) spectra of F1, F2, and F3 (**a**, **b**, and **c**, respectively) of collagen from skin hake.

The FTIR spectra revealed the characteristic peaks of amide A, B, I, II, and III of fibrillary collagen, in the three fractions obtained F1, F2, and F3, which were similar to those found in the collagen obtained from the skin of other fish species [30,31,32,33,34,35,36,37] (Figure 7). The helix is held together by hydrogen bonds from the glycine NH to the carbonyl residue of another strand. The N-H stretching vibration typical of intermolecular hydrogen bonding of a NH group with a carbonyl group of the neighboring peptide chain, commonly observed in the range 3000–3500 cm^−1^, corresponds to the Amide A band and was observed at 3293 cm^−1^, 3290 cm^−1^, and 3289 cm^−1^ for F1, F2, and F3, respectively. The Amide B (asymmetrical stretch of CH_2_) was observed in the region of 2927–2931 cm^−1^. The Amide I bands (polypeptide backbone stretching vibrations of carbonyl groups C=O, with characteristic frequencies in the range 1600–1700 cm^−1^) were found at 1633–1643–1630 cm^−1^ for F1, F2, and F3, respectively. The Amide II (N-H bending vibration coupled with C-N stretching vibration) bands were found at 1544–1546–1544 cm^−1^ for F1, F2, and F3, respectively, confirming the secondary structure [33]. The amide III band (C–H stretching) at 1238 cm^−1^ revealed that collagens did not denature during extraction and exhibited triple helical structure. Absorption intensity ratios between amide III and 1450 cm^−1^ were near 1.0, which is an indication of the preservation of the triple helix structure (ratio for denatured collagen, gelatin, is just about 0.6 according to Hu et al. 2014 [38]. The amide I, II, and III of F1 and F3 were found at lower wavenumbers, compared to F2, suggesting the presence of more or stronger hydrogen bonds in ASC than in PSC fractions—similar results to what has been also previously reported by Chen et al. [34] studying skin collagen from tilapia.

#### 3.4.4. Circular Dichroism

**Figure 8:**
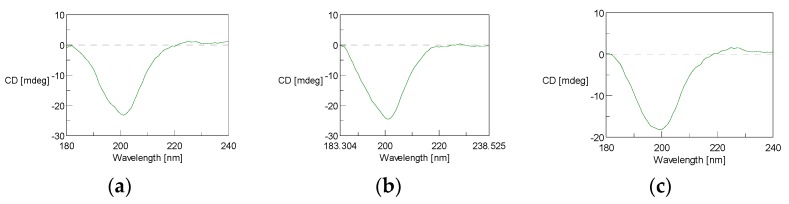
Circular Dichroism (CD) spectra of F1, F2, and F3 (**a**, **b**, and **c**, respectively) fractions of collagen from hake skin measured at 18 °C.

Circular dichroism (CD) measurements showed the characteristic CD spectrum of collagen (triple helical protein conformation), with a negative peak at around 200 nm, being the random coil structure, and a positive peak at around 225 nm, which is characteristic of triple helical protein conformation [39,40], for F1, F2, and F3 (Figure 8a–c). However, the weak positive peak observed for F2 (Figure 8b) indicates that, although present, the amount of triple helix for this fraction was lower than in the other fractions, as might be expected for a PSC fraction in which the collagen might be partially denatured. This result agrees with the lower intensity bands showed for *α*_1_ and *α*_2_ bands in fraction 2 compared to fractions 1 and 3 (ASC) in electrophoretic profiles (Figure 10). This result is also in accordance with results obtained in FTIR analysis. The positive *θ* (mdeg) values found for ASC fractions (F1 and F3) have also been found in other ASC collagen obtained from different marine species [6,41].

#### 3.4.5. X-ray Diffraction

**Figure 9:**
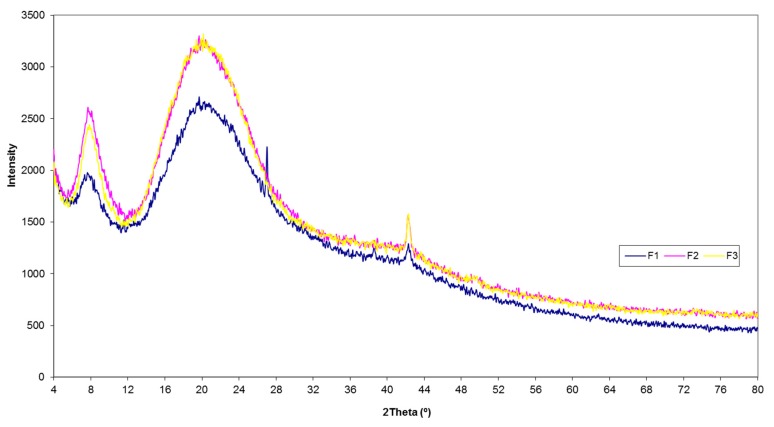
X-ray diffraction spectra of F1, F2, and F3 collagen from hake skin.

As shown in Figure 9, the XRD diagrams exhibit two peaks at diffraction angles of 7.62° and 20.21° (F1), 7.67° and 19.91° (F2), and 7.82° and 20.19° (F3), characteristic of collagen with native triple helix. The second peak was wider than the first one in the three fractions, which is in accordance with the characteristic diffraction peaks of collagen [29]). The Bragg equation was used to calculate the minimum value of the repeated spacings. The d values for the first and second peaks indicate the distance between molecular chains within the triple helix (i.e., it is related to the diameter of the triple helix chain) and the distance between skeletons (i.e., it is related to the diameter of the single left-handed helix chain), respectively. The d values obtained for F1, F2, and F3 were 11.59 Å and 4.39 Å, 11.51 Å and 4.45 Å, and 11.30 Å and 4.39 Å for the first and second peak, respectively. These results agree with d values reported for other collagens obtained from different marine species maintaining the triple helical structure [29,31,42].

#### 3.4.6. SDS-PAGE

**Figure 10:**
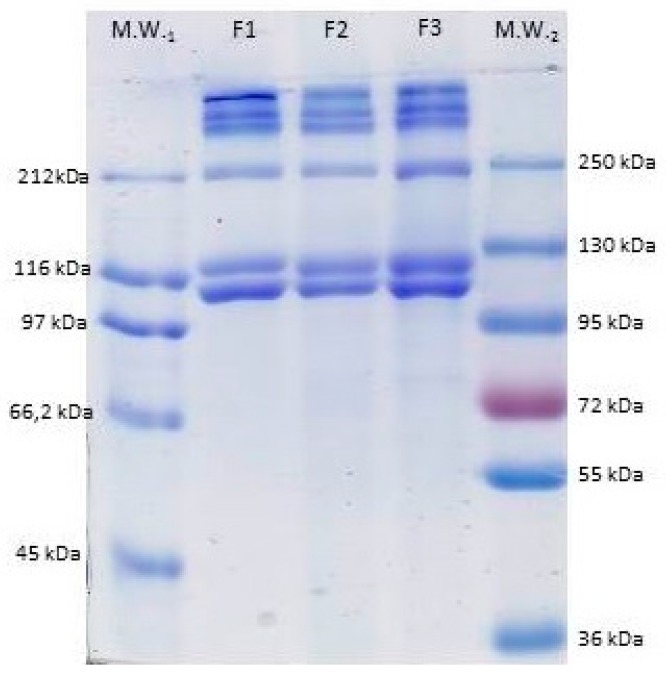
7% Sodium dodecylsulfate poly-acrylamide gel electrophoresis (SDS-PAGE) profile of F1, F2, and F3 of *M. merluccius* collagen. *M*_W_: molecular weight standards.

Electrophoretic patterns of the three collagen fractions obtained from the skin of *M. merluccius* were similar (Figure 10). ASC from the skin (F1) and bone (F3) were similar to one another and also compared to the PSC from the skin (F2), indicating that pepsin did not significantly affect the integrity of the triple helical structure. All fractions consisted of two *α*-chains (*α*1 and *α*2) with molecular weights of about 118 and 105 kDa. The band intensities of both *α*-chains were similar, with a slightly higher intensity corresponding to the band with the lower molecular weight. There was also some dimer (*β* chain) and trimer (*γ* chain) components formed by intramolecular and intermolecular cross-links of *α*-chains. The intensity of *β* and *γ* components was slightly higher in F1 and F3, indicating that ASC had more cross-linking than the F2 fraction (PSC), which is in accordance with other results regarding collagen extracted from different species [42].

## 4. Conclusions

As far as the authors know, this is the first time in which an extraction of collagen from a mixture of skin and bones obtained from a mechanical separation of muscle using fresh undersized specimens of *M. merluccius* has been studied. Skin and bone fraction obtained after the mechanical separation of the muscle using undersized specimens of *M. merluccius* represents an excellent raw material for the extraction of collagen. Three collagen fractions (ASC and PSC from the skin and ASC from the bones) were isolated from this raw material for the first time, with a total yield in a dried basis of 13.55% (g collagen/100 g of SBF) and 47.80% (g collagen/100 g of collagen determined by the hydroxyproline content in SBF). Physical and chemical characterization of collagen concluded to classify it as type I collagen. Althought PSC fraction (F2) seems to have fewer/weaker bonds compared to ASC fractions (F1 and F3), it contributes to a higher collagen recovery. This study presents a new approach to valorize subproducts that have originated from hake discards, which, under the LO, should not be transformed into edible products but other alternative applications. With the process herein presented, deboned and skinned hake specimens could represent an interesting source of high-quality type I collagen that could be used as a raw material in the biomedical, cosmetic, and nutraceutical industries. Future work will explore the possibilities of developing scaffolds with this collagen for the regeneration of tissues (bone and cartilage) and also for obtaining hydrolysates for cosmetics.

## Figures and Tables

**Table 1:** Amino acid composition of three collagen fractions (F1, F2, and F3) obtained from the skin and bones of *M. merluccius*.

Amino Acid	Collagen Fractions (nmols/mg)		
	F1	F2	F3
**OHpro**	416.31 ± 53	421.03 ± 148	440.01 ± 18
**Asp**	397.96 ± 22	455.76 ± 54	413.03 ± 40
**Thr**	225.60 ± 15	246.02 ± 36	232.79 ± 18
**Ser**	414.09 ± 26	463.12 ± 96	439.29 ± 45
**Glu**	577.27 ± 34	672.81 ± 79	604.96 ± 50
**Pro**	713.18 ± 47	750.22 ± 216	740.38 ± 31
**Gly**	2402.60 ± 211	2469.78 ± 701	2480.66 ± 27
**Ala**	920.25 ± 52	972.22 ± 242	925.32 ± 56
**Cys**	20.95 ± 3	24.87 ± 7	20.72 ± 4
**Val**	163.85 ± 16	177.08 ± 11	169.62 ± 10
**Met**	130.92 ± 6	138.62 ± 15	129.39 ± 14
**Ile**	79.78 ± 8	92.88 ± 10	84.71 ± 5
**Leu**	174.65 ± 7	216.24 ± 40	190.23 ± 26
**Tyr**	43.71 ± 9	62.04 ± 28	46.93 ± 24
**Phe**	123.56 ± 10	155.46 ± 36	130.66 ± 31
**Ohlys**	37.95 ± 5	56.61 ± 25	52.12 ± 17
**His**	63.14 ± 2	74.60 ± 14	67.69 ± 11
**Lys**	228.61 ± 6	262.17 ± 30	225.91 ± 25
**Arg**	368.93 ± 30	386.26 ± 92	380.43 ± 22

## References

[1] European Union (2013). Reglamento (UE) N° 1380/2013 Del Parlamento Europeo y Del Consejo De 11 De Diciembre De 2013.

[2] Blanco M., Sotelo C.G., Pérez-Martín R.I. (2015). Hydrolysis as a valorization strategy for unused marine food biomass: Boarfish and small-spotted catshark discards and by-products. J. Food Biochem..

[3] Blanco M., Vázquez J.A., Pérez-Martín R.I., Sotelo C.G. (2017). Hydrolysates of Fish Skin Collagen: An Opportunity for Valorizing Fish Industry Byproducts. Mar. Drugs.

[4] Vázquez J.A., Blanco M., Massa A.E., Amado I.R., Pérez-Martín R.I. (2017). Production of Fish Protein Hydrolysates from *Scyliorhinus canicula* Discards with Antihypertensive and Antioxidant Activities by Enzymatic Hydrolysis and Mathematical Optimization Using Response Surface Methodology. Mar. Drugs.

[5] Vázquez J.A., Fernández-Compás A., Blanco M., Rodríguez-Amado I., Moreno H., Borderías J., Pérez-Martín R.I. (2019). Development of bioprocesses for the integral valorisation of fish discards. Biochem. Eng. J..

[6] Alves A.L., Marques A.L.P., Martins E., Silva T.H., Reis R.L. (2017). Cosmetic potential of marine fish skin collagen. Cosmetics.

[7] Venkatesan J., Anil S., Kim S.-K., Shim M.S. (2017). Marine Fish Proteins and Peptides for Cosmeceuticals: A Review. Mar. Drugs.

[8] Gómez-Guillén M.C., Turnay J., Fernández-Díaz M.D., Ulmo N., Lizarbe M.A., Montero P. (2002). Structural and physical propoerties of gelatin extracted from different marine species: A comparative study. Food Hydrocoll..

[9] Karayannakidis P.D., Chatziantoniou S.E., Zotos A. (2014). Effects of selected process parameters on physical and sensorial properties of yellowfin tuna (*Thunnus albacares*) skin gelatin. J. Food Process Eng..

[10] Sotelo C.G., Blanco M., Ramos-Ariza P., Pérez-Martín R.I. (2016). Characterization of collagen from different discarded fish species of the west coast of the Iberian Peninsula. J. Aquat. Food Prod. Technol..

[11] Blanco M., Vázquez J.A., Pérez-Martín R.I., Sotelo C.G. (2019). Collagen extraction optimization from teh skin of the small-spotted catshark (*S. canicula*) by Response Surface Methodology. Mar. Drugs.

[12] Zhu B.W., Dong X.P., Zhou D.Y., Gao Y., Yang J.F., Li D.M., Zhao X.K., Ren T.T., Ye W.X., Tan H. (2012). Physicochemical properties and radical scavenging capacities of pepsin-solubilized collagen from sea cucumber *Stichopus japonicus*. Food Hydrocoll..

[13] Woo J.W., Yu S.J., Cho S.M., Lee Y.B., Kim S.B. (2008). Extraction optimization and properties of collagen from yellowfin tuna (*Thunnus albacares*) dorsal skin. Food Hydrocoll..

[14] Benjakul S., Thiansilakul Y., Visessanguan W., Roytrakul S., Kishimura H., Prodpran T., Meesane J. (2010). Extraction and characterisation of pepsin-solubilised collagen from the skin of bigeye snapper (*Priacanthus tayenus* and *Priacanthus macracanthus*). J. Sci. Food Agric..

[15] Muralidharan N., Shakila R.J., Sukumar D., Jeyasekaran G. (2013). Skin, bone and muscle collagen extraction from the trash fish, leather jacket (*Odonus niger*) and their characterization. J. Food Sci. Technol..

[16] Mahboob S. (2015). Isolation and characterization of collagen from fish waste material-skin, scales and fins of *Catla catla* and *Cirrhinus mrigala*. J. Food Sci. Technol..

[17] EU, DG MARE (2018). The EU Fish Market 2018.

[18] European Commission (2011). Impact assessment of discard reducing policies. Studies in the Field of the Common Fisheries Policy and Maritime Affairs.

[19] Valeiras J., Pérez N., Araujo H., Salinas I., Bellido J.M. (2014). Atlas De Los Descartes De La Flota De Arrastre u Enmalle En El Caladero Nacional Cantábrico-Noroeste.

[20] AOAC (2000). Official Methods of Analysis of the AOAC.

[21] Bligh E.G., Dyer W.J. (1959). A rapid method of total lipid extraction and purification. Can. J. Biochem. Phys..

[22] Meyer M., Morgenstern B. (2003). Characterization of gelatin and acid soluble collagen by size exclusion chromatography coupled with multi angle light scattering. Biomacromolecules.

[23] Laemmli U.K. (1970). Cleavage of Structural Proteins during the Assembly of the Head of Bacteriophage T4. Nature.

[24] Ministerio de Agricultura, Pesca y Alimentación (1995). Real Decreto 560/1995, De 7 De Abril, Por El Que Se Establece Las Tallas Mínimas De Determinadas Especies Pesqueras.

[25] Borges L. (2017). One Year on: The Landing Obligation in Europe. News ICES.

[26] Chef’s Resources. http://www.chefs-resources.com/seafood/seafood-yields/.

[27] Edwards C.A., O’Brien W.D. (1980). Modified assay for determination of hydroxyproline in a tissue hydrolysate. Clin. Chim. Acta.

[28] Carbalho A.M., Marques A.P., Silva T.H., Reis R.L. (2018). Evaluation of the potential of collagen from codfish skin as a biomaterial for biomedical applications. Mar. Drugs.

[29] Zhang F., Wang A., Li Z., He S., Shao L. (2011). Preparation and characterization of collagen from freshwater fish scales. Food Nutr. Sci..

[30] Ahmad M., Benjakul S., Nalinalon S. (2010). Composition and physicochemical characteristics of acid solubilized collagen extracted from the skin of unicorn leatherjacket (*Aluterus Monoceros*). Food Hydrocoll..

[31] De Sousa R.A.O. (2017). Valorization of Cod (*Gadus morhua*) by-Products by Extraction of Collagen with Potential Application in Cosmetic and Biomedical Field. Ph.D. Thesis.

[32] Habermehl J., Skopinska J., Boccafoschi F., Sionkowska A., Kaczmarek H., Laroche G., Mantovani D., Skopiǹska J. (2005). Preparation of Ready-to-use, Stockable and Reconstituted Collagen. Macromol. Biosci..

[33] Veeruraj A., Arumugam M., Balasubramanian T. (2013). Isolation and characterization of thermostable collagen from the marine eel-fish (*Evenchelys macrura*). Process Biochem..

[34] Chen J., Ruizao L.L., Xu N., Gao R., Hong B. (2016). Extraction and characterization of acid-soluble collagen from scales and skin of tilapia (*Oreochromis niloticus*). LWT Food Sci. Technol..

[35] Lee J.K., Kang S.I., Kim Y.J., Kim M.J., Heu M.S., Choi B.D. (2016). Comparison of collagen characteristics of sea-and freshwater rainbowtrout skin. Food Sci. Biotechnol..

[36] Ramanathan G., Singarevelu S., Raja M., Sobhana S., Sivagranam U.T. (2014). Extraction and characterization of collagen from the skin of *Arotron stellatus* fish—A novel source of collagen for tissue engineering. J. Biomater. Tissue Eng..

[37] Riaz T., Zeeshan R., Zarif F., Ilyas K., Nawshad M., Safi S.Z., Rahim A., Rizvi S.A.A., Rehman I.U. (2018). FTIR analysis of natural and synthetic collagen. Appl. Spectrosc. Rev..

[38] Hu Y., Liu L., Gu Z., Dan W., Dan N., Yu X. (2014). Modification of collagen with a natural derived cross-linker, alginate dialdehyde. Carbohyd. Polym..

[39] Drzewieccki K.E., Grisham D.R., Parmar A.S., Nanda V., Shreiber D.I. (2016). Circular dichroism spectroscopy of collagen fibrillogenesis: A new use for an old technique. Biophys. J..

[40] Greenfield N.J. (2006). Using circular dichroism spectra to estimate protein secondary structure. Nat. Protoc..

[41] Fiorani A., Gualandi C., Panseri S., Montesi M., Marcacci M., Focarete M., Bigi A. (2014). Comparative performance of collagen nanofibers electrospun from different solvents and stabilized by different crosslinkers. J. Mater. Sci. Mater. Med..

[42] Sun L., Li B., Song W., Si L., Hou H. (2017). Characterization of Pacific cod (*Gadus macrocephalus*) skin collagen and fabrication of collagen sponge as a good biocompatible biomedical material. Process Biochem..

